# Lessons learned from Vietnam's first COVID-19 vaccine rollout: tackling vaccine hesitancy and misinformation for future pandemic responses

**DOI:** 10.3389/fpubh.2025.1633756

**Published:** 2025-10-17

**Authors:** Ngoc Minh Tam Bui, Hong Trang Nguyen, Bich Thuy Duong, Tri Nugraha Susilawati, Truc Thanh Thai, Minh Thuy Vu, Jialing Lin, Thuy Anh Bui, Minh Cuong Duong

**Affiliations:** ^1^University Medical Center Ho Chi Minh City, Ho Chi Minh City, Vietnam; ^2^Faculty of Nursing, Phenikaa University, Hanoi, Vietnam; ^3^Infectious Diseases Department, FV Hospital, Ho Chi Minh City, Vietnam; ^4^Faculty of Medicine, Universitas Sebelas Maret, Surakarta, Indonesia; ^5^Department of Medical Statistics and Informatics, University of Medicine and Pharmacy at Ho Chi Minh City, Ho Chi Minh City, Vietnam; ^6^International Centre for Future Health Systems, University of New South Wales, Sydney, NSW, Australia; ^7^School of Population Health, Faculty of Medicine & Health, University of New South Wales, Sydney, NSW, Australia

**Keywords:** COVID-19 vaccine, hesitancy, information channels, infodemic, lessons, pandemic preparedness

## Abstract

**Background:**

Vaccine hesitancy and misinformation significantly undermine pandemic preparedness. Insights from the COVID-19 vaccine rollout can inform and enhance future pandemic responses. During Vietnam's Delta outbreak in 2021, the vaccine rollout faced significant challenges including limited vaccine supply and public vaccine hesitancy, which impeded widespread coverage.

**Objectives:**

This study examined levels of COVID-19 vaccine hesitancy, common information sources, exposure to negative vaccine-related information, and intentions to promote vaccination among the Vietnamese public.

**Methods:**

A national cross-sectional survey was conducted in 2021 with 1,579 participants recruited through the snowball sampling method. Logistic regression analyses were used to identify factors associated with vaccine hesitancy.

**Results:**

Overall, 30.6% of respondents were vaccine hesitators. Hesitancy was significantly higher among females [adjusted Odds Ratio (AOR) = 1.438, 95% CI: 1.132–1.892, *P* = 0.003), non-health students (AOR = 1.924, 95% CI: 1.017–3.642, *P* = 0.044), non-health workers (AOR = 2.168, 95% CI: 1.293–3.636, *P* = 0.003), those with a high school education (AOR = 2.073, 95% CI: 1.365–3.147, *P* = 0.001) or below (AOR = 2.722, 95% CI: 1.143–6.486, *P* = 0.024). Lower hesitancy was associated with prior paid vaccination (AOR = 0.718, 95% CI: 0.56–0.92, *P* = 0.009), and good vaccine knowledge (AOR = 0.484, 95% CI: 0.382–0.613, *P* < 0.001). Social media (76%), peers (65.9%), television news (63.6%), and newspapers (62.8%) were common information sources. Notably, 89% encountered negative or misleading information, and only 47.8% were willing to promote vaccination.

**Conclusions:**

Addressing vaccine hesitancy in future outbreaks of COVID-19 and other infectious diseases requires combating misinformation, especially on social media, and improving vaccine knowledge among lower-education groups. Individuals in health-related sectors and those who have previously paid for vaccinations can serve as role models in promoting vaccination. Clear, culturally appropriate communication and sustained government are vital to counter the infodemic, build trust, and improve vaccine uptake in future pandemics. Due to snowball sampling, the study findings may not generalize to rural, older, or less-educated populations. Future studies should consider stratified sampling to improve representativeness.

## Introduction

The COVID-19 pandemic has posed an unprecedented global health challenge since late 2019 with high early global mortality rates especially in vulnerable populations and health systems under strain (1). Vaccination quickly emerged as the most effective strategy to reduce infection, hospitalizations, mortality, and healthcare costs, even in countries with relatively low disease burden (2, 3). The first COVID-19 vaccines were administered in December 2020, and sustaining high levels of vaccine uptake and booster acceptance remains vital for preventing future outbreaks (2). However, vaccine rollout has varied widely across nations, influenced by disparities in resources, healthcare infrastructure, and public trust (4). While high-income countries generally achieved rapid vaccine deployment, many low- and middle-income countries (LMICs) faced delays due to limited access and logistical barriers (4).

Vietnam presents a distinctive case among LMICs within the global context of the COVID-19 pandemic. Initially praised for its swift containment efforts in 2020, the country was hit hard by the Delta variant in mid-2021, with over 1.3 million cases and nearly 28,000 deaths (5, 6). Hospitals in major cities, particularly those in densely populated urban areas and resource-limited regions, faced overwhelming demand (6). Vaccines first became available in March 2021, with rollout expanding quickly to reach over 70% of the population by the end of 2021 (7). Despite this progress, vaccine hesitancy, mainly driven by misinformation, safety concerns, and varying levels of trust, led to delays in uptake, especially in rural and underserved communities (7). As a result, during the peak of the Delta wave, over 600,000 cases and 15,000 deaths occurred before improved coverage helped contain the outbreak (6).

Reflecting on Vietnam's COVID-19 response, it becomes clear that achieving broader and faster vaccine coverage, alongside addressing vaccine hesitancy, was crucial for controlling virus transmission, limiting case surges, and preventing avoidable deaths (8). Although COVID-19 vaccination rates in Vietnam improved by late 2021, only around 50% of the population was fully vaccinated by October, highlighting the need for more rapid and widespread immunization (7). To enhance future pandemic responses, it is essential to identify demographic and socio-economic groups prone to vaccine hesitancy and to understand the factors driving their reluctance (7). Such insights will support the creation of tailored, culturally sensitive interventions to boost vaccine acceptance, strengthening disease control efforts in Vietnam and other LMICs with similar challenges.

This study investigates COVID-19 vaccine hesitancy during Vietnam's initial vaccine rollout, particularly among unvaccinated adults. It explores the sociodemographic characteristics associated with hesitancy, common sources of vaccine information, exposure to negative or misleading content, and individuals' willingness to recommend vaccination. The study also explores public knowledge, beliefs, and attitudes toward COVID-19 vaccines, identifying key gaps that influence vaccine acceptance. Understanding these factors can provide valuable insights to develop targeted, culturally appropriate public health strategies. The findings aim to support Vietnam and other LMICs facing similar challenges in strengthening public trust, countering misinformation, and improving vaccine uptake in preparation for future infectious disease outbreaks.

## Methods

### Study context

A population-based cross-sectional survey was conducted across Vietnam between 16 April and 16 July 2021, during the country's first significant COVID-19 outbreak. By the end of the study period, a total of 40,609 cumulative incident cases had been documented, affected 52.4% (33 out of 63) cities across Vietnam (9). Ho Chi Minh City, one of the two main research sites, was the epicenter of the outbreak, accounting for 58.9% (23,913 out of 40,609) of all cumulative incident cases (10). During this time, Vietnam began its initial COVID-19 vaccine rollout, although uptake remained limited. Notably, a few vaccine-related deaths were reported in Vietnam during this period, which contributed to public anxiety and emerging concerns about vaccine safety (11).

### Study design

#### Questionnaire

A self-administered questionnaire was developed and included two sections: participant demographics and the COVID-19 vaccine-related items (Supplementary Appendix S1). The demographic section comprised 11 questions, including year of birth, gender, residential address, living arrangement, level of education, occupations, Gapminder income classification (12), presence of chronic conditions, prior experience with COVID-19, and history of receiving vaccines outside the Expanded Program on Immunization (i.e., paid vaccines). The COVID-19 vaccine section included (1) a Likert scale question assessing participants' willingness to receive the COVID-19 vaccine once available, with five response options (strongly agree, agree, neutral/no opinion, disagree, strongly disagree), (2) seven Likert scale questions assessing knowledge about COVID-19 vaccines and vaccination, (3) two yes-no questions regarding COVID-19 vaccination status and any direct or indirect negative experience with vaccines, including COVID-19 vaccine, that may discourage them from getting the COVID-19 vaccine, and (4) four multiple-choice questions identifying sources of COVID-19 vaccine-related information, exposure to negative or misleading content, and willingness to recommend vaccination to others.

For analysis, those who responded “strongly agree” or “agree” to the COVID-19 vaccine acceptance question were categorized as vaccine acceptors, and those selecting “neutral/no opinion,” “disagree,” or “strongly disagree” were considered vaccine hesitators. Regarding seven vaccine knowledge questions, each item had five response options, scored from 0 to 4, corresponding to increasing levels of agreement or disagreement with the statement. For positively phrased items (items 3, 4, 6, and 7), “strongly disagree” was scored as 0, whereas for negatively phrased items (items 1, 2, and 5), “strongly agree” was scored as 0. Negatively phrased items were reverse-coded so that higher scores consistently reflected greater knowledge. The maximum possible total knowledge score was 28. The items were designed to avoid leading questions and to ensure balanced distribution of responses. All questionnaire items were developed based on the existing literature, published information from the vaccine manufacturers, and guidance from the WHO and Vietnam Ministry of Health at the time of the study (3, 10, 13).

#### Data collection

Two data collection methods were used: offline (paper-based) and online surveys. For the offline approach, participants were provided with a printed participant information sheet, completed a written informed consent form and filled out a paper version of the questionnaire. For the online survey, the paid SurveyMonkey platform (http://www.surveymonkey.com/) was utilized. Participants first reviewed an electronic participant information and consent page, then responded to a yes-no question confirming their voluntary consent to participate. Upon confirmation, they were directed to complete the online questionnaire. This approach to obtaining informed consent has been validated elsewhere (14).

Within the participant information sheet, participants were clearly informed of the study's objectives, confidentiality protocols, and eligibility criteria. Upon completion of data collection, participants' year of birth was used to verify age, and only data from individuals aged 18 and older were included in the final analysis. Since vaccine uptake may reflect acceptance, it is an important factor when estimating vaccine acceptance rates. However, to better inform COVID-19 vaccination policies aimed at increasing coverage, our study specifically focused on individuals who were unvaccinated at the time of data collection. Consequently, respondents who had already received a COVID-19 vaccine before the survey were excluded from the analysis. In addition, to remove duplicate responses in the online survey, submissions from identical Internet Protocol (IP) address were manually reviewed by two researchers (MCD and HTN), and only one entry was retained following consensus.

#### Recruitment of study participants

The snowball sampling method was used to recruit participants for both online and offline surveys. Recruitment relied on the authors' professional and personal networks, which included individuals from diverse backgrounds such as healthcare professionals, university lecturers, students, and members of the general community. A recruitment poster and a survey link were disseminated via the authors' groups and timelines on widely used social media platforms in Vietnam, such as LinkedIn, Zalo, and Facebook as well as through email to their professional and personal networks. Recipients were encouraged to further share the poster and the survey with their own networks to expand reach. Offline data collection was conducted in Ho Chi Minh City and Hanoi, the two largest cities and major internal migration hubs in Vietnam. The selection of these sites enabled the inclusion of participants from a wide range of socioeconomic and geographic backgrounds across the country.

#### Refining the questionnaire

Pilot surveys were conducted both online and offline to refine the final questionnaire and assess its validity and reliability. Each pilot included 50 study participants recruited from diverse backgrounds. Responses and participant feedback were systematically analyzed to identify ambiguities and improve question clarity. This approach to validation has been adopted in previous research (15). To ensure participant comprehension, the survey was administered in Vietnamese for both online and offline formats. Contact details of the researchers (MCD and HTN) were also provided, allowing participants to seek clarification or assistance if needed.

#### Ethical approval

The study was performed in accordance with the ethical principles of the Declaration of Helsinki and was approved by the Phenikaa University Ethics Committee (reference 216/QĐ-ĐHP-KHCN). All study participants provided informed consent.

### Statistical analysis

Data were analyzed using IBM Statistical Package for Social Sciences (SPSS) version 26. Data were presented as mean ± standard deviation for continuous variables and number (percentage) for categorical variables. The proportion of vaccine hesitancy and 95% confidence interval (95% CI) were calculated. The distribution of categorical variables was examined and, where appropriate, combined categories with small counts to ensure stable estimation while preserving conceptual meaning.

In this study, participants' vaccine knowledge levels were categorized using a cut-off value equal to the mean score of all participants. Given that the individual's score is a whole number, if the cut-off value is a decimal number, a “good” vaccine knowledge level will be defined as an individual's score higher than or equal to the nearest whole number higher than the cut-off value, while an individual's score lower than the nearest whole number indicates a “poor” level. If the cut-off value is a whole number, a “good” level will be defined as an individual's score higher than the cut-off value, while an individual's score lower than or equal to the cut-off value indicates a “poor” level. This analysis approach is validated elsewhere (16). Group comparisons for continuous variables were performed using the student *T* test. Categorical variables were compared using the Chi-squared or Chi-squared test for trend, as appropriate. A binary logistic regression analysis was developed to identify factors independently associated with vaccine hesitancy. All relevant independent variables were included in the binary logistic regression model to comprehensively assess factors associated with the outcome. These variables included demographic characteristics (e.g., age, gender, education level, and Gapminder-classification-based daily income groups), chronic conditions, experience with COVID-19, history of receiving a paid vaccine, any previous adverse vaccine experiences, and COVID-19 vaccine knowledge. Statistical significance (alpha) was set at *P* < 0.05.

### Maintenance of the study standard

To enhance the transparency of the study and facilitate interpretation of the findings, this study adheres to the Checklist for Reporting Results of Internet E-Surveys (CHERRIES) (17) and the Strengthening the Reporting of Observational Studies in Epidemiology (STROBE) guidelines for Observational Studies (18).

## Results

### Demographics

**Figure 1:**
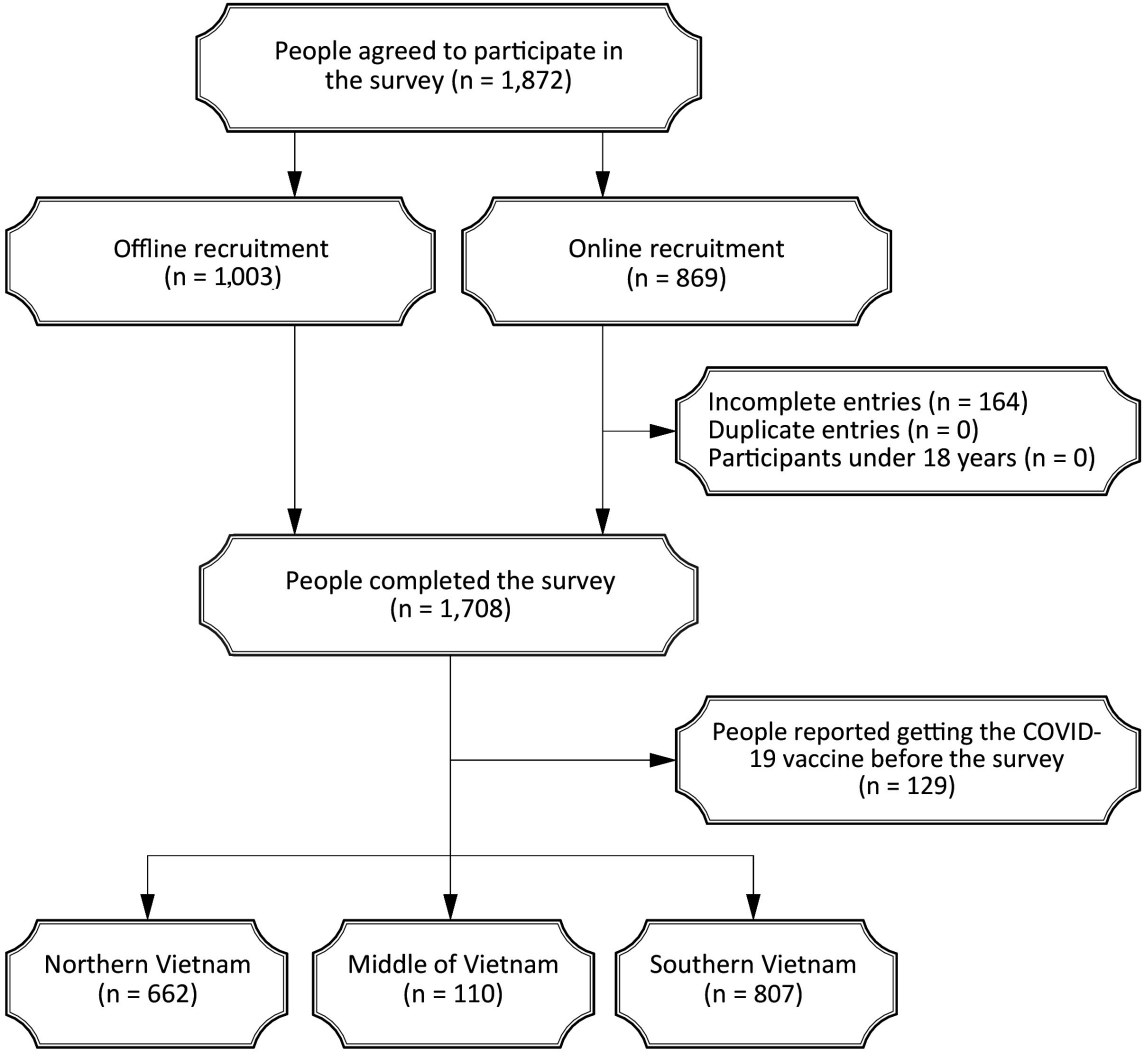
Flow chart of study participants.

A total of 1,872 participants were recruited, including 1,003 (53.6%) through offline approach and 869 (46.4%) online (Figure 1). Of these, 164 online participants (8.8%) did not complete the survey, and 129 (6.9%) reported having received a COVID-19 vaccine prior to participation. These individuals were excluded from the final analysis. Consequently, the study comprised 1,579 responses from participants representing all three regions of Vietnam (Table 1). The mean age was 34 (±13.7) years, and females accounted for 54.5% (860/1,579) of the sample. Most respondents were either students or those employed in non-health related fields (76.6%, 1,210/1,579), held a bachelor's degree or higher (81.1%, 1,280/1,579), earned less than USD$15 per day (70%, 1,106/1,579), and lived with their family (72.9%, 1,151/1,579). Among them, 12.9% (203/1,579) reported chronic health conditions, 2.6% (41/1,579) had experienced COVID-19, 67.9% (1,072/1,579) had previously received paid vaccines, and 44.3% (699/1,579) reported negative experiences related to vaccination. Given that the mean vaccine knowledge score of all participants was 19.2 ± 2.8 (Supplementary Appendix S2), 46.4% (732/1,579) demonstrated good knowledge of COVID-19 vaccines.

**Table 1:** Baseline characteristics of 1,579 participants.

**Variables**	All participants^*^
**Age (years)**	34 ± 13.7 (18.0–80.0)
**Female**	860 (54.5)
**Residency location in Vietnam**	
The North	662 (41.9)
The South	807 (51.1)
The Middle	110 (7.0)
**Gapminder-classification-based daily income groups (USD$)**	
< 2	343 (21.7)
2– < 8	230 (14.6)
8– < 15	533 (33.8)
15–32	307 (19.4)
>32	166 (10.5)
**Housing**	
Living alone	130 (8.2)
Living with relatives	1,151 (72.9)
Living with friends/colleagues	298 (18.9)
**Levels of education**	
Below high school	29 (1.8)
High school	138 (8.7)
College	132 (8.4)
Bachelor's degree level or above	1,280 (81.1)
**Occupations**	
Health professions students	120 (7.6)
Non-health professions students	378 (23.9)
Non-health related workers	832 (52.7)
Other health related workers	128 (8.1)
Physicians and/or health lecturers	121 (7.7)
**Chronic conditions** ^a^	203 (12.9)
**Experience with COVID-19** ^b^	41 (2.6)
**Taking paid vaccine(s) previously** ^c^	1,072 (67.9)
**Having bad experience with vaccines** ^d^	699 (44.3)
**Having good COVID-19 vaccine knowledge**	732 (46.4)

^*^Continuous data are reported as “mean ± 1 standard deviation (minimum – maximum),” while categorical data are presented as “absolute count (percentage)”.

^a^Chronic conditions include communicable and non-communicable diseases.

^b^Acquiring COVID-19 disease oneself and/or having relative(s) or friend(s)/colleague(s) with COVID-19.

^c^Taking vaccine(s) that was(were) not integrated into the Vietnam's national Expanded Program on Immunization.

^d^Experiencing directly and/or indirectly vaccine related event(s) including that of COVID-19 vaccines that may discourage oneself from getting a COVID-19 vaccine.

### Prevalence of COVID-19 vaccine hesitancy, vaccine information channels and willingness to support vaccination

Vaccine hesitancy was reported by 30.6% (483/1,579, 95% CI: 28.4%−32.9%) of participants (Table 2). The most common sources of vaccine information were social media (76%, 1,200/1,579), friends or colleagues (65.9%, 1,040/1,579), and mainstream media including television news (63.6%, 1,004/1,579), newspapers (62.8%, 991/1,579) and official websites (36.3%, 573/1,579).

**Table 2:** COVID-19 vaccine hesitancy, information channels, acquired negative information, and willingness to promote vaccination among 1,579 participants.

**Characteristics**	***N* (%)**
**Vaccine hesitancy**	483 (30.6)
**Information channels**	
**Social media**	1,200 (76.0)
**Mainstream media and Vietnam government agency websites**	
TV news	1,004 (63.6)
Newspapers	991 (62.8)
Government websites	573 (36.3)
**Word of mouth**	
Friends/colleagues	1,040 (65.9)
Family members	743 (47.1)
**Health professionals**	
Doctors/nurses	174 (11)
Pharmacists	41 (2.6)
**Foreign websites**	374 (23.7)
**Having heard about negative information** ^*^	1,405 (89)
Negative information^*^(***n*** **=** **1, 405)**	
Vaccine causes deaths	914 (65.1)
Vaccine causes other side effects	647 (46.1)
Vaccine is not effective	405 (28.8)
Vaccine causes COVID-19 infection	95 (6.8)
**Willingness to encourage people to get a COVID-19 vaccine**	
Yes	755 (47.8)
No	303 (19.2)
Not sure	521 (33)

^*^COVID-19 vaccine related information that may discourage study participants from getting a COVID-19 vaccine.

The majority of participants (89%, 1,405/1,579) reported exposure to negative information that could discourage vaccination. The most prevalent themes included fears that the vaccine causes death (65.1%, 914/1,405) and other side effects (46.1%, 647/1,405), is ineffective (28.8%, 405/1,405), or could even cause COVID-19 infection (6.8%, 95/1,405). Fewer than half of participants (47.8%, 755/1,579) indicated willingness to encourage others to get vaccinated, while 33% (521/1,579) were unsure and 19.2% (303/1,579) expressed unwillingness.

### Predictors of COVID-19 vaccine hesitancy

In the univariable analysis, vaccine hesitancy was significantly associated with gender (*P* = 0.001), Gapminder income level (*P* = 0.039), education (*P* < 0.001), occupation (*P* = 0.028), previous receipt of paid vaccine(s) (*P* < 0.001), and having good COVID-19 vaccine knowledge (*P* < 0.001; Table 3). There was no significant association between vaccine hesitancy and age, region of residence, living arrangement, experience with COVID-19, and having bad experience with vaccines (*P* > 0.05).

**Table 3:** Predictors of COVID-19 vaccine hesitancy among 1,579 participants.

**Variables**	**Univariable analysis** ^**&**^				**Logistic regression analysis** ^**&**^	
	**COVID-19 vaccination** ^*^		**OR (95% CI)**	***P-*** **value**		
	**Hesitators (*****n*** = **483)**	**Acceptors (*****n*** = **1,096)**			**Adjusted OR (95% CI)**	***P-*** **value**
**Age (years)**	33.9 ± 13.7	34 ± 13.6	–	0.895^a^	–	0.611
**Female**	293 (60.7)	567 (51.7)	1.44 (1.16–1.79)	0.001^b^	1.438 (1.132–1.892)	0.003
**Residency location in Vietnam**						
The North	222 (46)	440 (40.1)	–	0.075^b^	–	0.125
The South	233 (48.2)	574 (52.4)				0.5
The Middle^**^	28 (5.8)	82 (7.5)				–
**Gapminder-classification based on daily income (US Dollar)**						
< 2	117 (24.2)	226 (20.6)	–	0.039^c^	–	0.68
2– < 8	84 (17.4)	146 (13.3)				0.968
8– < 15	155 (32.1)	378 (34.5)				0.262
15–32	86 (17.8)	221 (20.2)				0.785
>32^**^	41 (8.5)	125 (11.4)				–
**Housing**						
Living alone	33 (6.8)	97 (8.9)	–	0.404^b^	–	0.494
Living with relatives	358 (74.1)	793 (72.4)				0.504
Living with friends/colleagues^**^	92 (19.1)	206 (18.7)				–
**Levels of education**						
Below high school	18 (3.7)	11 (1)	–	< 0.001^c^	2.722 (1.143–6.486)	0.024
High school	61 (12.6)	77 (7)			2.073 (1.365–3.147)	0.001
College	42 (8.7)	90 (8.2)			–	0.343
Bachelor's degree level or above^**^	362 (74.9)	918 (83.8)				–
**Occupations**						
Health professions students	35 (7.2)	85 (7.8)	–	0.028^b^	–	0.257
Non-health professions students	127 (26.3)	251 (22.9)			1.924 (1.017–3.642)	0.044
Non-health related workers	264 (54.7)	568 (51.8)			2.168 (1.293–3.636)	0.003
Other health related workers	34 (7)	94 (8.6)			–	0.362
Physicians and/or health lecturers^**^	23 (4.8)	98 (8.9)				–
**Chronic conditions** ^**d**^	72 (14.9)	131 (12)	–	0.121^b^	–	0.181
**Experience with COVID-19** ^**e**^	12 (2.5)	29 (2.6)	–	1^b^	–	0.646
**Taking paid vaccine(s) previously** ^**f**^	294 (60.9)	778 (71)	0.64 (0.51–0.8)	< 0.001^b^	0.718 (0.560–0.920)	0.009
**Having bad experience with vaccines** ^**g**^	212 (43.9)	487 (44.4)	–	0.87^b^	–	0.523
**Having good COVID-19 vaccine knowledge**	166 (34.4)	566 (51.6)	0.49 (0.39–0.61)	< 0.001^b^	0.484 (0.382–0.613)	< 0.001

COVID-19, coronavirus disease 2019; CI, confidence interval; OR, odds ratio.

^*^Continuous data are reported as “mean ± 1 standard deviation (minimum–maximum),” while categorical data are presented as “absolute count (percentage).”

^**^Reference group in regression analysis.

^a^t-test.

^b^Chi-squared test.

^c^Chi-squared test for trend.

^d^Chronic conditions include communicable and non-communicable diseases.

^e^Acquiring COVID-19 disease oneself and/or having relative(s) or friend(s)/colleague(s) with COVID-19.

^f^Taking vaccine(s) that was(were) not integrated into the Vietnam's national Expanded Program on Immunization.

^g^Experiencing directly and/or indirectly vaccine related event(s) including that of COVID-19 vaccines that may discourage oneself from getting a COVID-19 vaccine.

In the multivariable logistic regression analysis, being female was independently associated with increased odds of vaccine hesitancy [adjusted OR (AOR) = 1.438, 95% CI: 1.132–1.892, *P* = 0.003]. In contrast, previous receipt of paid vaccine(s) (AOR = 0.718, 95% CI: 0.56–0.92, *P* = 0.009) and having good COVID-19 vaccine knowledge (AOR = 0.484, 95% CI: 0.382–0.613, *P* < 0.001) were associated with lower odds of vaccine hesitancy. Compared to participants with a bachelor's degree or higher, those with only a high school education (AOR = 2.073, 95% CI: 1.365–3.147, *P* = 0.001) or below high school education (AOR = 2.722, 95% CI: 1.143–6.486, *P* = 0.024) had significantly higher odds of vaccine hesitancy. Additionally, being a non-health student (AOR = 1.924, 95% CI: 1.017–3.642, *P* = 0.044) or working in a non-health related occupation (AOR = 2.168, 95% CI: 1.293–3.636, *P* = 0.003) had significantly higher odds of vaccine hesitancy compared with physicians and/or health lecturers.

## Discussion

Vaccination is one of the most effective and cost-effective public health interventions to prevent mortality and disability caused by infectious diseases. Maintaining optimal levels of vaccine uptake and acceptance, particularly for booster doses, remains essential to prevent future outbreaks of COVID-19 and other similar infectious diseases (1, 2). This study represents one of the largest surveys on COVID-19 vaccine hesitancy in Vietnam and provide critical insights into the factors influencing public acceptance during a severe outbreak. The age and gender distribution of our participants closely mirrored national census data (19), strengthening the representativeness of our findings.

Our main finding is that despite the severity of the epidemiological situation at the time, only 69.4% of participants reported an intention to be vaccinated against COVID-19. While this figure exceeds the commonly cited 60% threshold for effective pandemic control (20), it still reflects a concerning level of vaccine hesitancy. This rate is comparable to findings from a study of 2,695 individuals across five major cities in the United States, United Kingdom, and Australia, which reported vaccine acceptance ranging from 70.5 to 77.5% depending on the city (21). In contrast, our rate was substantially higher than the 17% acceptance observed in a large multinational Arabic-speaking cohort (15), but lower than the 76.4% reported in a study conducted across nine other LMICs (22). These discrepancies may reflect differences in local pandemic severity, sociopolitical contexts, and levels of trust in public health authorities at the time of data collection. Importantly, individuals' perceptions of COVID-19 risk and vaccine safety have been consistently shown to influence their willingness to be vaccinated (15, 23–25). Despite a moderate overall acceptance rate, only 47.8% of respondents in our study were willing to actively advocate for vaccination. This limited advocacy likely reflects barriers identified in our concurrent qualitative research, including concerns about side effects, doubts about vaccine effectiveness against emerging variants, practical challenges such as cost and access, and a preference for vaccination at well-equipped sites (26). Similar concerns have been observed in other Vietnamese studies among health science students and families hesitant to vaccinate their children (27, 28). These findings highlight the complexities involved in introducing new public health interventions during the emergence of a pandemic. Public hesitation toward COVID-19 vaccines during the early rollout in 2021 was understandable, especially following temporary suspensions of the AstraZeneca vaccine in several countries and precautionary advisories for the Moderna vaccine due to rare but serious side effects (29–31). Addressing vaccine hesitancy therefore requires a thorough understanding of public concerns (32) and clear communication of scientific evidence to improve vaccine acceptance (32).

Given the importance of promoting vaccine literacy, a key question is how effectively we have communicated with our communities. Our study explored how respondents accessed and utilized various information channels to obtain vaccine-related information. Social media was the most cited source (76%) followed by over 60% relying on mainstream media such as television and newspapers. In contrast, engagement with healthcare professionals was minimal, suggesting that many individuals may be left vulnerable to misinformation or lack access to trusted, evidence-based guidance (33). In this context, 89% of our participants reported encountering negative or fear-inducing vaccine content, contributing to concerns that vaccines cause death (65.1%), serious side effects (46.1%), or are ineffective (28.8%). Notably, a 35-year-old medical worker in An Giang province died from anaphylactic shock after receiving the AstraZeneca vaccine in May 2021, marking Vietnam's first reported and extremely rare vaccine-related death (34). Such fears, often driven by online misinformation and conspiracy narratives (35), likely undermine confidence and willingness to promote vaccination (36). Nevertheless, given the powerful influence of digital media, it is essential that researchers, healthcare professionals, and policymakers actively utilize these communication platforms to monitor public sentiment (33), understand the underlying reasons for vaccine refusal (37), closely monitor adverse events following immunization (38) and incorporate these insights into the design and implementation of future public health interventions (39). As Larson et al. (32) emphasize, one key strategy to strengthen vaccine advocacy is to engage healthcare professionals as vaccine champions, trusted figures who can understand patient concerns, provide reassurance, and recommend appropriate interventions (32). This approach is particularly relevant in our context, where engagement with healthcare professionals was notably low.

Another important aspect to consider is why misinformation triggers behavioral responses such as vaccine hesitancy. Psychological mechanisms, such as confirmation bias and fear appeals, can significantly influence how individuals process and interpret information, reinforcing existing concerns and skepticism about vaccines (36). These mechanisms mirror broader trends documented in the literature on the COVID-19 infodemic, defined as the rapid spread of false or misleading information that undermines public health efforts by fueling confusion and distrust (40). Given the prominence of social media as an information source and its potential to propagate harmful content, these findings highlight an urgent need to strengthen media literacy and public education (35). In Vietnam, as in many other countries, authorities faced major challenges in countering misinformation during the early stages of the pandemic (41). In response, the government launched official Ministry of Health communication channels via Zalo and Facebook in late 2020, delivering timely and credible information directly to the public (41). These efforts played a key role in achieving high vaccine uptake, with over 70% of the population fully vaccinated by December 2021 (42). This experience suggests that equipping individuals with reliable and accessible sources of information is essential to mitigating the behavioral impacts of the infodemic and fostering informed vaccine decision-making.

Our findings highlight that vaccine hesitancy is shaped by key sociodemographic factors, including gender, education level, and professional background. The observed gender differences in vaccine acceptance align with findings from several international studies, which reported mixed outcomes: some identified higher hesitancy among women (15, 22, 23, 43), while others found greater hesitancy among men (44). Our concurrent qualitative research suggests that vaccine hesitancy among women may be shaped by sociocultural factors, including concerns about vaccine safety during pregnancy or breastfeeding, potential effects on fertility, and caregiving responsibilities that increase perceived risks to themselves and their families (26). Previous research suggests that women often exhibit greater caution toward novel vaccines, potentially due to heightened sensitivity to safety concerns or exposure to misinformation (45, 46). Additionally, prevailing social and cultural norms surrounding health decision-making and risk perception may further contribute to gender-based disparities in vaccine acceptance (47). Beyond gender, our findings align with regional studies showing that individuals with lower education, rural residence, economic disadvantage, unemployment, and residence in Vietnam or Indonesia were more likely to report COVID-19 vaccine hesitancy (14, 48). Those without a health-related background, particularly non-health students and workers, were less likely to express vaccination intent. A mid-2021 study in Vietnam similarly found that hesitancy among health professions students stemmed from concerns about vaccine safety, severe side effects, and a lack of transparent information (14, 49). In contrast, positive media messaging, prior influenza vaccination, and greater perceived risk of COVID-19 were linked to lower hesitancy (49). Together, these findings underscore the importance of tailored, trust-building communication strategies that address specific demographic and professional concerns.

Beyond sociodemographic factors, psychological determinants might also play a crucial role in vaccine hesitancy. Related studies conducted on the same participant cohort highlight the significant influence of psychological and cultural factors. Our initial study found that trust was highest in government officials and physicians, but lowest in religious leaders, underscoring the essential role of trusted recommenders in promoting vaccine acceptance (50). Furthermore, our qualitative investigation revealed dynamic vaccine attitudes shaped by individuals' risk–benefit self-assessments and a strong public trust in authorities, emphasizing the importance of perceived risk and culturally embedded trust in government-led vaccination campaigns (26). These findings suggest that addressing vaccine hesitancy requires not only targeting sociodemographic disparities but also strengthening trust and delivering culturally appropriate, clear communication to effectively counter misinformation and promote vaccine uptake.

A notable observation from our study was the positive association between prior experience with paid vaccines and COVID-19 vaccine acceptance. It is plausible that individuals who have previously paid for vaccinations perceive them as valuable and are thus more inclined to accept newer vaccines (24). However, interpreting this association requires further contextualization within the Vietnamese immunization landscape. In Vietnam, paid immunization programs operate alongside the government-funded Expanded Program on Immunization (EPI), offering access to vaccines not included in the national schedule, such as influenza, pneumococcal, zoster, and HPV vaccines (51). These paid vaccines vary in their recommended use, with some targeted at specific subgroups, such as older adults or immunocompromised individuals. Therefore, assuming uniform exposure to or uptake of paid vaccines across all demographic groups may result in unintentional stratification and bias when examining their relationship with COVID-19 vaccine attitudes (52). Despite this complexity, our findings are consistent with previous studies showing that individuals socialized into a pro-vaccination culture, through routine or voluntary vaccination, tend to carry these attitudes into adulthood (15, 23–25).

The findings from this study can be contextualized within the Health Belief Model (HBM), which posits that individuals' engagement in health behaviors, such as vaccine uptake, is influenced by perceived susceptibility to the disease, perceived severity, perceived benefits of vaccination, perceived barriers, cues to action, and self-efficacy (53). In our study, greater knowledge about COVID-19 vaccines likely reduced perceived barriers and increased perceived benefits, contributing to lower hesitancy. This observation is consistent with findings from China (54) and Indonesia (55). Conversely, exposure to misinformation, particularly via social media, may have heightened perceived risks or barriers, undermining confidence and trust in vaccines. Our observation contrasts with findings from Bangladesh, where respondents who accessed vaccine information via social media (e.g., Facebook), online news portals, or blogs were found to be less hesitant (56). These findings suggest that trust in official sources may serve as an important cue to action, reinforcing individuals' intentions to vaccinate and promote vaccination to others. Future interventions could leverage these HBM constructs to develop more targeted, theory-informed communication strategies tailored to specific population contexts.

Our study has several limitations. The snowball sampling technique and the online nature of our survey, while practical during the peak of the outbreak and strict self-isolation measures in Vietnam, may have introduced sampling bias. Given that the study used two survey methods, performing sensitivity analyses to determine if the two samples differed in any sociodemographic characteristics and responses would add more insights into the study findings. However, this was overlooked in our study. Although the age and gender distribution in our sample were comparable to national data (19), participants likely shared the survey within their own networks, resulting in over-representation of younger, healthier respondents with only 12.9% reporting chronic health conditions, urban, highly educated with 81.1% holding a bachelor's degree or higher, and digitally connected individuals. We acknowledge that individuals with strong opinions about vaccines may have been more motivated to participate, while those without internet access or sufficient digital literacy were less likely to be included. Although we attempted to address this by supplementing offline recruitment in two major cities, the sample still likely under-represents key populations such as rural residents, older adults, high-risk groups, and those with lower educational levels. Furthermore, results for subgroups with small sample sizes should be interpreted with caution, as limited statistical power may increase the risk of unstable estimates. These findings are therefore considered exploratory, and larger, more representative studies are recommended to confirm associations in these specific populations.

We also acknowledge that the majority of our respondents were non-health workers or students, which limits the insights we can draw regarding perspectives within the health sector. However, a previous study conducted among health students in Vietnam around the same time as our study has provided valuable complementary data on vaccine attitudes and hesitancy in the health sector (27). Additionally, grouping “neutral/no opinion” with “disagree” responses may obscure nuanced differences in attitudes but was done to capture overall hesitancy during a critical period of widespread misinformation. These limitations might restrict the generalizability of our findings to the broader Vietnamese population. A further limitation is the use of the sample mean score as the sole cut-off to classify vaccine knowledge levels. While this approach provided a simple and interpretable reference point in the absence of an established benchmark, alternative thresholds were not examined. As such, our findings should be interpreted with caution, and future studies should explore whether different cut-off points yield consistent results.

Moreover, we also acknowledge that our model excluded local vaccine availability and economic status, which may affect hesitancy and should be considered in future studies. As vaccine attitudes and access can vary significantly across sociodemographic groups, future studies should consider employing probability sampling such as stratified random methods (57) to ensure more representative inclusion across age, education, and geographic location. Additionally, statistical weighting adjustments could be used to correct for sampling imbalances and improve population-level inferences (58). In addition, the timing of the survey presents another limitation, as attitudes toward COVID-19 vaccination are likely to evolve in response to changes in epidemiological trends, vaccine availability, and public messaging (59). Notably, our qualitative study conducted around the same time found that COVID-19 vaccine attitudes in Vietnam were mixed and dynamic, shaped by individual risk-benefit assessments and level of trust in external sources (26). Future research should also consider employing longitudinal method to monitor shifts in vaccine attitudes over time.

In conclusion, our findings underscore that a considerable proportion of the Vietnamese public remained hesitant to receive COVID-19 vaccines even amid a major outbreak. This hesitancy reflects underlying barriers, including concerns about vaccine safety and the influence of misinformation. Addressing these challenges requires multifaceted strategies to strengthen public trust in vaccines through accurate, targeted communication campaigns; improve access to credible vaccine information; and encourage positive vaccination experiences. Government leadership is indispensable in combating misinformation and building vaccine confidence. Understanding the barriers and enablers identified in this study will be crucial for shaping future efforts to improve COVID-19 booster uptake and to enhance preparedness for future infectious disease pandemics, not only in Vietnam but also in similar LMIC contexts.

## Data Availability

The raw data supporting the conclusions of this article will be made available by the authors, without undue reservation.
