# Supplementary material for: Lessons learned from Vietnam's first COVID-19 vaccine rollout: tackling vaccine hesitancy and misinformation for future pandemic responses
Source: Front Public Health. 2025 Oct 17;13:1633756. doi: 10.3389/fpubh.2025.1633756 (PMC12575300; doi:10.3389/fpubh.2025.1633756)
Supplement: Supplementary file 1 [file Data_Sheet_1.docx]

**Questionnaire**

1. **General information:**

| 1. Year of birth (please specify): | ………. |
| --- | --- |
| 1. Gender: | □ Male □ Female |
| 1. Residential address (please only specify the city name): | …………. |
| 1. Region of residence: | □ Northern Vietnam □ Middle of Vietnam  □ Southern Vietnam |
| 1. Gapminder income levels (US$ per day)*: | □ <2  □ 2 - <8  □ 8 - ≤15  □ 15 - <32  □ ≥32 |
| 1. Living arrangement: | □ Living alone  □ Living with family  □ Living with friends |
| 1. Level of education: | □ Less than high school  □ High school  □ College  □ Undergraduate level or above |
| 1. Occupations: | □ Health students  □ Non-health students  □ Working in non-health related fields  □ Being clinical doctor and/or health lecturer  □ Working in other health related fields |
| 1. Chronic conditions: | □ Do not have chronic conditions  □ Having chronic, noncommunicable diseases  □ Having chronic, communicable diseases |
| 1. Experience with COVID-19: | □ Having COVID-19 or acquired COVID-19 previously  □ Having a family member who has COVID-19 or acquired COVID-19 previously  □ Having a friend/colleague who has COVID-19 or acquired COVID-19 previously  □ Never acquire COVID-19, or know anyone who has COVID-19 or acquired COVID-19 previously |
| 1. Receiving vaccine(s) outside the Expanded Program on Immunization | □ Yes  □ No |

*To assist study participants in completing the questionnaire easily, the currency was converted to VND and the unit was VND per month in the Vietnamese version of the questionnaire.

1. **COVID-19 vaccine knowledge and acceptance:**

**COVID-19 vaccine acceptance:**

**I accept COVID-19 vaccine if generally available**

| Strongly  agree | Agree | Neutral/no opinion | Disagree | Strongly disagree |
| --- | --- | --- | --- | --- |
|  |  |  |  |  |

**COVID-19 vaccine knowledge:**

1. **I am completely protected against COVID-19 after I fully complete the COVID-19 vaccination schedule**

| Strongly  agree | Agree | Neutral/no opinion | Disagree | Strongly disagree |
| --- | --- | --- | --- | --- |
|  |  |  |  |  |

1. **I do not need to undertake any other COVID-19 preventive measures after I fully complete the COVID-19 vaccination schedule**

| Strongly  agree | Agree | Neutral/no opinion | Disagree | Strongly disagree |
| --- | --- | --- | --- | --- |
|  |  |  |  |  |

1. **Being vaccinated for COVID-19 myself contributes to the protection of the community against COVID-19**

| Strongly  agree | Agree | Neutral/no opinion | Disagree | Strongly disagree |
| --- | --- | --- | --- | --- |
|  |  |  |  |  |

1. **Getting vaccinated for COVID-19 is a good way to protect myself from COVID-19**

| Strongly  agree | Agree | Neutral/no opinion | Disagree | Strongly disagree |
| --- | --- | --- | --- | --- |
|  |  |  |  |  |

1. **I do not need to get vaccinated for COVID-19 because the COVID-19 outbreak is controlled very well in Vietnam.**

| Strongly  agree | Agree | Neutral/no opinion | Disagree | Strongly disagree |
| --- | --- | --- | --- | --- |
|  |  |  |  |  |

1. **COVID-19 vaccines developed by different manufacturers have different levels of efficacy**

| Strongly  agree | Agree | Neutral/no opinion | Disagree | Strongly disagree |
| --- | --- | --- | --- | --- |
|  |  |  |  |  |

1. **The available COVID-19 vaccines may be less effective on new variants compared with the original strain**

| Strongly  agree | Agree | Neutral/no opinion | Disagree | Strongly disagree |
| --- | --- | --- | --- | --- |
|  |  |  |  |  |

**COVID-19 vaccination status and negative vaccine experience:**

| 1. Do you remember any vaccine related events in the past and COVID-19 vaccine related events that may discourage you from getting the COVID-19 vaccines for yourself? | □ Yes  □ No |
| --- | --- |
| 1. Have you received a COVID-19 vaccine | □ Yes  □ No |

1. **Vaccine – information channels**
2. **Which of the followings are your sources of information about COVID-19 vaccine? (check all if appropriate)**

| □ TV news  □ Social media  □ Newspapers | □ Friends/ colleagues  □ Family members  □ Pharmacists | □ Doctors/ nurses  □ Government websites  □ Foreign websites |
| --- | --- | --- |

1. **Have you ever heard any negative information regarding COVID-19 vaccine that may discourage you from getting the vaccine?**

Yes □ No □ (please ignore questions 2.1)

**2.1. Which of the followings are the negative information about COVID-19 vaccine that you heard? (check all if appropriate)**

|  Vaccine causes deaths   Vaccine causes COVID-19 infection |  Vaccine causes other side effects   Vaccine is not effective in protecting against COVID-19 infection |
| --- | --- |

1. **Are you willing to encourage people to get vaccinated for COVID-19?**

Yes □ No □ Not sure □
