# Supplementary material for: Lessons learned from Vietnam's first COVID-19 vaccine rollout: tackling vaccine hesitancy and misinformation for future pandemic responses
Source: Front Public Health. 2025 Oct 17;13:1633756. doi: 10.3389/fpubh.2025.1633756 (PMC12575300; doi:10.3389/fpubh.2025.1633756)
Supplement: Supplementary file 2 [file Data_Sheet_2.docx]

**Appendix 2.** **Detail of participants’ responses to seven COVID-19 vaccine knowledge questions and mean knowledge score**

| **Questions response**  **(assigned score)** | **Summary statistics**  **n (%)** |
| --- | --- |
| **Q1. I am completely protected against COVID-19 after I fully complete the COVID-19 vaccination schedule** | |
| Strongly agree (0) | 262 (16.6) |
| Agree (1) | 689 (43.6) |
| Neutral/no opinion (2) | 368 (23.3) |
| Disagree (3) | 228 (14.4) |
| Strongly disagree (4) | 32 (2.0) |
| **Q2. I do not need to undertake any other COVID-19 preventive measures after I fully complete the COVID-19 vaccination schedule** | |
| Strongly agree (0) | 22 (1.4) |
| Agree (1) | 52 (3.3) |
| Neutral/no opinion (2) | 197 (12.5) |
| Disagree (3) | 739 (46.8) |
| Strongly disagree (4) | 569 (36.0) |
| **Q3. Being vaccinated for COVID-19 myself contributes to the protection of the community against COVID-19** | |
| Strongly agree (4) | 465 (29.4) |
| Agree (3) | 849 (53.8) |
| Neutral/no opinion (2) | 223 (14.1) |
| Disagree (1) | 30 (1.9) |
| Strongly disagree (0) | 12 (0.8) |
| **Q4. Getting vaccinated for COVID-19 is the best way to protect myself from COVID-19** | |
| Strongly agree (4) | 325 (20.6) |
| Agree (3) | 811 (51.4) |
| Neutral/no opinion (2) | 306 (19.4) |
| Disagree (1) | 124 (7.9) |
| Strongly disagree (0) | 13 (0.8) |
| **Q5. I do not need to get vaccinated for COVID-19 because the COVID-19 outbreak is controlled very well in Vietnam** | |
| Strongly agree (0) | 15 (0.9) |
| Agree (1) | 27 (1.7) |
| Neutral/no opinion (2) | 198 (12.5) |
| Disagree (3) | 670 (42.4) |
| Strongly disagree (4) | 669 (42.4) |
| **Q6. COVID-19 vaccines developed by different manufacturers have different levels of efficacy** | |
| Strongly agree (4) | 268 (17.0) |
| Agree (3) | 750 (47.5) |
| Neutral/no opinion (2) | 443 (28.1) |
| Disagree (1) | 99 (6.3) |
| Strongly disagree (0) | 19 (1.2) |
| **Q7. The available COVID-19 vaccines may be less effective on new variants compared with the original strain** | |
| Strongly agree (4) | 204 (12.9) |
| Agree (3) | 805 (51.0) |
| Neutral/no opinion (2) | 521 (33.0) |
| Disagree (1) | 43 (2.7) |
| Strongly disagree (0) | 6 (0.4) |
| **Mean knowledge score** | |
| Knowledge score (mean ± SD (min – max)) | 19.2 ± 2.8 (11 - 28) |
